# On the Origin of Tibetans and Their Genetic Basis in Adapting High-Altitude Environments

**DOI:** 10.1371/journal.pone.0017002

**Published:** 2011-02-28

**Authors:** Binbin Wang, Yong-Biao Zhang, Feng Zhang, Hongbin Lin, Xumin Wang, Ning Wan, Zhenqing Ye, Haiyu Weng, Lili Zhang, Xin Li, Jiangwei Yan, Panpan Wang, Tingting Wu, Longfei Cheng, Jing Wang, Duen-Mei Wang, Xu Ma, Jun Yu

**Affiliations:** 1 National Research Institute for Family Planning, Beijing, People's Republic of China; 2 Graduate School, Peking Union Medical College, Beijing, People's Republic of China; 3 CAS Key Laboratory of Genome Sciences and Information, Beijing Institute of Genomics, Chinese Academy of Sciences, Beijing, People's Republic of China; 4 Technology Division of CID Regiment, Public Security Department, Lhasa, People's Republic of China; 5 World Health Organization Collaborating Centre for Research in Human Reproduction, Beijing, People's Republic of China; King Abdullah University of Science and Technology, Saudi Arabia

## Abstract

Since their arrival in the Tibetan Plateau during the Neolithic Age, Tibetans have been well-adapted to extreme environmental conditions and possess genetic variation that reflect their living environment and migratory history. To investigate the origin of Tibetans and the genetic basis of adaptation in a rigorous environment, we genotyped 30 Tibetan individuals with more than one million SNP markers. Our findings suggested that Tibetans, together with the Yi people, were descendants of Tibeto-Burmans who diverged from ancient settlers of East Asia. The valleys of the Hengduan Mountain range may be a major migration route. We also identified a set of positively-selected genes that belong to functional classes of the embryonic, female gonad, and blood vessel developments, as well as response to hypoxia. Most of these genes were highly correlated with population-specific and beneficial phenotypes, such as high infant survival rate and the absence of chronic mountain sickness.

## Introduction

Humans first reached the Tibetan Plateau during the Last Glacial Maximum (22–8 kya) [1], and modern Tibetans can be traced back to Neolithic immigrants based on evidence found in the Y chromosome [2] and mitochondrial DNA [3]. However, the exact origin of modern Tibetans has been widely debated due to varying and conflicting evidence from archaeology, historical records, linguistics, and genetics [3], [4]. Previous studies have suggested, based on genetic evidence, two distinct possibilities for whom the ancestors of modern Tibetans were: people who lived in the upper and middle Yellow River basin [3], [5] and Northern Asian populations [6]. A suspected migration route for the Tibetans' ancestors was the so-called “Zang (meaning Tibetan people) - Yi (the Yi people) - Corridor” which supposed that Tibetans first migrated from Qinghai to the Tibetan Plateau and then subsequently spread throughout the surrounding area [7].

The Tibetan Plateau is unique in its high absolute elevation and low temperature. However, Tibetans have lived on the plateau for tens of thousands of years and adapted to the high-altitude environment better than other populations. Tibetans exhibit many biological features in common with other high-altitude mammalian species (such as antelopes and pigs), including absence of chronic mountain sickness (CMS), thin-walled pulmonary vascular structure, and high blood flow [8]; all these phenotypes are highly correlated with physiological responses to low oxygen concentration in the air, which facilitate uninterrupted oxygen-processing and the up-regulation of erythropoiesis and angiogenesis to allow for more efficient oxygen utilization.

Human adaptation to high-altitude environment is believed to a result of advantageous genetic mutation and selective pressure. Many well-characterized human genes that play important roles in environmental adaptation have been identified, such as *HBB* (*Hemoglobin-B*), which causes resistance to malaria, and *LCT* (*lactase*), which is essential for the digestion of dairy products [9]. Similarly, genes that participate in the physiological response to hypoxia may also be excellent indicators of adaptation. This idea is supported by two lines of evidence. First, Tibetans have distinctive biological characteristic – elevated resting ventilation, which offsets the huge stress of hypoxia [10]. Second, Tibetans have been exposed to hypoxia for about 1,100 generations [1] when enough time has passed for an increase in the frequency of adaptive alleles to be fixed [10].

Three recent studies have identified several genes that play important roles in high-altitude adaptation, including *EGLN1*, *PPARA*, and *EPAS1* [11], [12], [13]. However, these studies have not been entirely adequate. In two of the three studies, the Tibetan samples or part of them were collected in Qinghai Province [12] or Yunnan Province [11], but not Tibet itself. Meanwhile, samples are admixture with 2 [13] or 3 [11] geographic locations. Furthermore, none of the studies provided information concerning migration or ancestry. To investigate genetic signatures for the origin of Tibetans, and search for genes involved in high altitude adaptation, we genotyped 30 Tibetan individuals from pasture areas near Lhasa (3700 meters in altitude) with Illumina Human-1M chips. The resulting genotypic data was analyzed along with data sets from HapMap and HGDP.

## Results

### Population genetic analyses

**Figure 1:**
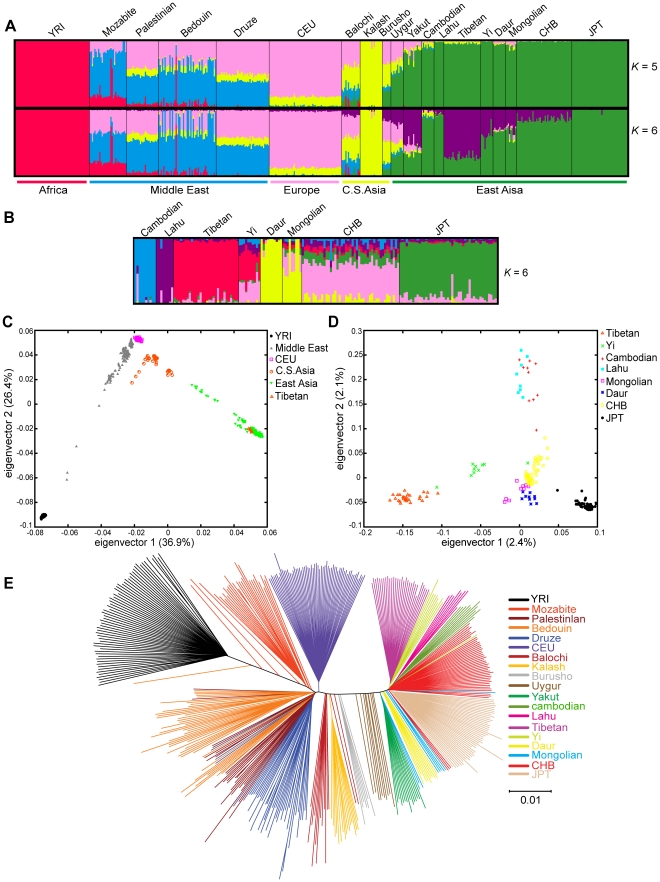
Genetic relationships between population pairs of Tibetan and others. (A) The ancestry sharing proportion of 497 individuals (from the 19 world-wide populations) inferred with *frappe* at *K* = 5 and *K* = 6, using 165,073 loosely linked autosomal SNPs. Each vertical line represents an individual and is composed of colored segments whose lengths represent the individual's coefficients in *K* speculated ancestral groups. (B) The ancestry sharing proportion of 167 individuals (from the 8 EA populations) at *K* = 6, using 165,073 autosomal SNPs. (C, D) Principal component analyses of population structure on the 19 worldwide populations (C) and the 8 East Asian populations (D), using 509,491 autosomal SNPs. (E) Neighbor-joining phylogenic tree of the 497 individuals of 19 world-wide populations, using 165,073 autosomal SNPs. The color of each individual was assign according to their population affiliation. C.S. Asia: South/Central Asia, YRI: Yoruban in Ibadan, CHB: Han Chinese in Beijing, JPT: Japanese in Tokyo.

To investigate the genetic relationship between Tibetans and other populations, we analyzed our Tibetan genotype data in conjunction with data from HapMap (Phase II) and HGDP (Human Genome Diversity Project). Nineteen world-wide populations (497 individuals) and ten East Asian (EA) populations (192 individuals) were included in the metadata. 509,491 autosomal SNPs overlapped within this dataset (Table S1). We used 165,073 less-linked SNPs (r^2^<0.5) from the dataset to perform individual ancestry and admixture proportions analysis, assuming a range of ancestral components from 2 to 6 (*K* = 2 to 6) without prior knowledge concerning population identity (Figure 1 and Figure S1).

In the case of the world-wide dataset, the results were similar to those reported by Li et al [14]—individual genetic populations remained tightly correlated to their geographic locations and virtually every population had only a single inferred ancestral component. After the incorporation of the Tibetan data, we observed a new ancestral component arisen predominantly from the Tibetans, which divided the EA populations into two new groups other than the well-defined Northern and Southern groups (Figures 1A and S1B) [14]. At *K* = 2, we had two ancestral components: the Tibetan and Japanese ancestries, while from *K* = 3 to 6, the Southern (Cambodian and Lahu), Northern (Mongolian, Daur, and CHB which is Chinese Han from HapMap), CHB, and Lahu populations appeared accordingly. At *K* = 6, we had the Tibetan, Cambodian, Lahu, Daur, and JPT (Japanese from HapMap) populations, and each exhibited only one major component in its ancestry. In sharp contrast, there were the Yi, Mongolian, and Han populations (represented by CHB); all had multiple ancestries (Figure 1B). In short, Tibetans appeared to share the majority of their ancestry with EA populations.

To capture the major directions of genetic variation, we performed principal component analyses (PCA) on both world-wide and EA populations at the individual level based on genotypic information from 509,491 SNPs. The PCA plot for world-wide samples showed that populations within a continental/regional group were clearly separated from one another (Figure 1C). The dispersal of individuals in the plot is consistent with the process of population expansion posited by the “out of Africa” theory. Tibetans are clustered within EA populations, in agreement with the results of ancestry analysis (Figure 1D). The first eigenvector shows the divergence between Tibetans and Japanese within EA populations. The second eigenvector shows the Northern and Southern distinction. The third eigenvector distinguishes Mongolian and Daur from CHB in the Northern EA populations, and the fourth eigenvector distinguishes Cambodian from Lahu in the Southern EA populations (Figure S2). The closest population to Tibetans is the Yi, whose genetic variability has contributions from both Tibetans and Han Chinese (Figure 1D).

We constructed an unrooted neighbor-joining phylogenetic tree based on the distance matrix of nucleotide information from 165,073 poorly-linked SNPs (Figure 1E). The populations that were dominated by one major ancestry in our earlier analysis, such as CEU (descendant of European), Kalash, and Tibetans, have only one branch connecting to the tree trunk. In contrast, populations with admixed ancestries, especially for Middle-East and South/Central Asian populations, have many branches connecting to the trunk. Overall variance can be divided into three types of genetic variation: variation among-individuals-within-populations, among-populations-within-groups (i.e., geographical region), and among-groups. Within-population variation accounts for most of the genetic distance. Within- and among-group variation, however, was sufficient to reveal population structure; individuals with the same population identity were always clustered together, while those with different identities were well-separated. Therefore, large numbers of unique loci with subtle allele frequency changes yielded an accumulated effect for distinguishing each ethnic group. Inclusion of the Tibetan and Yi populations in the EA branch of the tree suggested that these two populations not only shared some genetic compositions but may also have used similar migration routes.

### Selection tests

To uncover the genetic imprints of harsh environmental factors over thousands of years, we investigated genetic differentiation between Tibetans and their geographic relatives—the EA populations. Since genetic loci with unusual degrees of differentiation often provide indications of selection [15], we used the outlier approach to detect positive selection. This is supported by previous findings that 60% of genes with extreme levels of population differentiation have undergone positive selection [16] and that this positive selection was strong enough to generate extreme spatial patterns compared to the rest of the genome [17].

We calculated the locus-by-locus pairwise *F*_ST_ between the Tibetan population and those from HapMap and HGDP (EA) under various SNP densities. To reduce locus-to-locus stochastic variation, we generated a test statistics from a 200-kb window as the average *F*_ST_ above a cutoff value [18]. Because population differentiation among continents is largely influenced by asymmetric gene flow and migration history, the contribution of selection to high *F*_ST_ is very limited [19]. As a result, in continental population pairs, only four well-characterized genes, *SLC24A5*, *SLC45A2*, *EDAR*, and *PAWR*, were positively selected (Figure S3). The genomic regions that were under positive selection in Tibetans were very similar to those of CHB and JPT. Since Tibetan and EA populations share a common ancestry, most genetic differentiation between them may be ascribed to local adaptation [15], [20]. We have shown those chromosomal regions that had extremely high *F*_ST_ among EA populations in Figure S4. We found that the most significant candidate locus under positive selection in Tibetan-contained pairs was the region containing *EPAS1* (*HIF-2α*) (*P*<0.001 in all population pairs with Tibetans).

**Figure 2:**
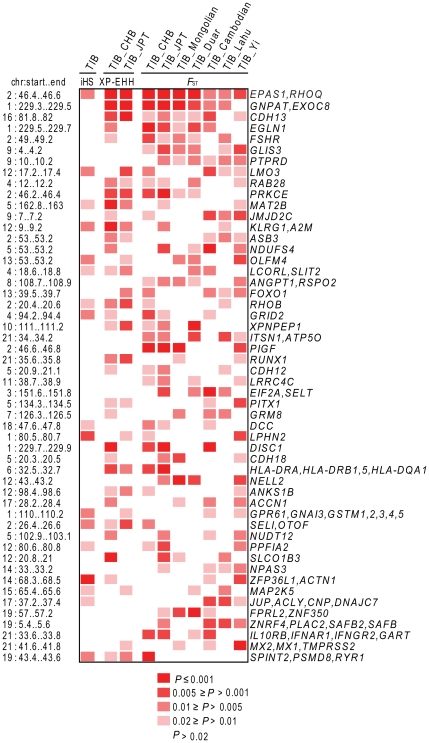
Significant genomic regions indentified in Tibetans by iHS, XP-EHH, and *F*_ST_. Ten selection tests (one iHS, two XP-EHH, and seven *F*_ST_, showed as columns) were performed on Tibetans or Tibetan-included pairs, and empirical *P*-value of each 200-kb genomic window (showed as row) was obtained. Windows with *P*≤0.02 are listed, and then sorted according to the numbers of significant appearances. Only windows that appeared at least four times are shown. The physical position of each window on the human genome is labeled on the left of plot. Genes within or near each window are shown on the right. Genes without functional summary provided by RefSeq were removed. The windows with no functional genes are not shown.

**Figure 3:**
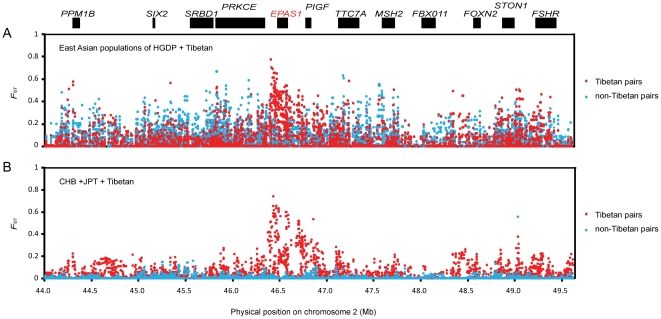
Pairwise *F*_ST_ values of the *EPAS1* gene and its surrounding regions. The plots of locus-by-locus pairwise *F*_ST_ of East Asian Populations of HGDP (including Yi, Mongolian, Duar, Lahu, and Cambodian) + Tibetan (A), and CHB, JPT, and Tibetan pairs (B) are shown. The x-axis of each plot is the physical position (Mb) on Chromosome 2. The y-axis of each plot is the value of pairwise *F*_ST_. Each dot of the plots represents a pairwise *F*_ST_ of a SNP. The extremely high *F*_ST_ are mostly located within the *EPAS1* gene.

Since genetic loci under positive selection may not always give rise to extremely high *F*_ST_ [19], we also used two haplotype-based methods, iHS (integrated Haplotype Score) and XP-EHH (Cross Population Extended Haplotype Homozygosity) to detect significant reductions in gene diversity around selected loci [21], [22], [23]. The most significant region (*P*<0.008 in all tests) was located on chromosome 2 from 46.4 Mb to 46.6 Mb; both of its adjacent 200-kb regions also showed significant values (Figure 2). This suggests that strong selection and the near-complete selective sweeps have occurred in this genomic region. To uncover the potential causal genes, we plotted the *F*_ST_ values of SNPs in a 6-Mb region flanking the site of interest, for *F*_ST_ signals normally peak around the causal variant [24]. In Figure 3, the *F*_ST_ signals peak at *EPAS1*, a critical hypoxia inducible factor, in all Tibetan pairs. For population pairs from other East Asian (including Yi), no peaks were observed at *EPAS1*. This suggests that *EPAS1* is potentially under positive selection only in Tibetans. The second significant region (*P*<0.02 in 6 tests) also show near-complete sweep to the surrounding 600-kb area. The *EGLN1* gene within this region is also involved in the response to hypoxia and potentially be the target of positive selection. *EPAS1* and *EGLN1* play central roles in the activation of hypoxia-inducible genes and homeostasis of HIF under hypoxia and normoxia [25], [26]. Other genomic regions yielded significant test statistics for selected genes, including *CDH13*, *ANGPT1*, *RUNX1*, *FOXO1*, *JMJD2C*, *GLIS3*, *MAT2B*, *A2M*, *RYR1*, and *NPAS3* (Figure 2).

Adaptation to high altitudes is a complex biological process and requires coordination among many genes and pathways. We took genes from the regions with pre-set cutoffs (*P*<0.05) of *F*_ST_, iHS, and XP-EHH for further functional analysis on GO terms (Table 1). We observed significant results in categories related to blood vessel development (*P* = 0.0064), response to hypoxia (*P* = 0.0096), embryonic development (*P* = 0.029), and female gonad development (*P* = 0.0196). The candidate genes, which include *EPAS1*, *ANGPT1*, *EGLN1*, *FOXO1*, *RUNX1*, *RYR1* and *CDH13,* may play essential roles in adaptation to high altitudes.

**Table 1:** GO term enrichment of candidate genes under positive selection.

GO term^a^	GO categroy	*P*-value^b^	Enrichment Score	Genes
GO:0001568	blood vessel development	0.0064	2.10	*EPAS1,ANGPT1,CDH13,FOXO1,WARS2,NRP2,LEPR,TGFBR3*
GO:0001944	vasculature development	0.0073		
GO:0001666	response to hypoxia	0.0096	1.99	*EPAS1,EGLN1,ANGPT1,RYR1,SLC8A1,ECE1*
GO:0070482	response to oxygen levels	0.0118		
GO:0048699	generation of neurons	0.0113	1.73	*AGTPBP1,DCC,LRRC4C,NRXN3,CNTNAP2,SLITRK6,CLN5,RUNX3,GNAT2,KLHL1,NRP2,PARD3*
GO:0022008	neurogenesis	0.0186		
GO:0048646	anatomical structure formation involved in morphogenesis	0.0188	1.70	*EPAS1,AGTPBP1,ANGPT1,CDH13,LEPR,TGFBR3,ZEB2,CELSR1,NRP2*
GO:0022602	ovulation cycle process	0.0180	1.62	*ANGPT1,FSHR,LEPR,PGR*
GO:0008585	female gonad development	0.0196		
GO:0046660	female sex differentiation	0.0238		
GO:0046545	development of primary female sexual characteristics	0.0238		
GO:0042698	ovulation cycle	0.0256		
GO:0043009	chordate embryonic development	0.0290	1.41	*EPAS1,EGLN1,ECE1,GRIN2B,TGFBR3,ZEB2,CELSR1,ACVR2A*
GO:0009792	embryonic development ending in birth or egg hatching	0.0428		
GO:0009887	organ morphogenesis	0.0295	1.40	*ANGPT1,GNPAT,SMARCD3,TGFBR3,PDGFC,ZEB2,CELSR1,MYC,WWOX,RUNX3,GNAT2*
GO:0048469	cell maturation	0.0296	1.39	*EPAS1,CLN5,RUNX3,PGR*
GO:0032504	multicellular organism reproduction	0.0418	1.36	*ANGPT1,FSHR,NPAS3,ARPL10L,PGR,ACVR2A,CYLC2,LEPR,TSNAX,QP7*
GO:0048609	reproductive process in a multicellular organism	0.0418		
GO:0008406	gonad development	0.0180	1.32	*ANGPT1,FSHR,LEPR,PGR,ACVR2A*
GO:0045137	development of primary sexual characteristics	0.0271		
GO:0048608	reproductive structure development	0.0335		

a: enriched GO terms in the subcategroy of biological process.

b: calculated by modified Fisher's exact test.

## Discussion

Our population genetic structure analyses suggested that Tibetans share the common ancestors with East Asian populations, but not Central/South Asian populations who settled on the western and southern side of Himalayas. Our finding is consistent with the results of a previous study which suggested gene-flow inhibition caused by the Himalayas [27]. We also showed that the closest relatives of the Tibetans are the Yi people, who live in the Hengduan Mountains and were originally formed through fusion with natives along their migration routes into the mountains [28]. The Tibetan and Yi languages belong to the Tibeto-Bruman language group and their ancestries can be traced back to an ancient tribe, the Di-Qiang [3], [28]. Both Tibetans and Yi are found in the same clade in the phylogenic tree, having emerged from ancient EA populations.

The migration routes of the Chinese population as a single group have been outlined based on Y chromosome haplotype distributions. After the ancestors of Sino-Tibetans reached the upper and middle Yellow River basin, they divided into two subgroups: Proto-Tibeto-Burman and Proto-Chinese [2]. These two subgroups were similar to the two ancestral components of EA populations at *K* = 2 (Figure S1B). The ancestral component which was dominant in Tibetan and Yi arose from the Proto-Tibeto-Burman subgroup, which marched on to south-west China and later, through one of its branches, became the ancestor of modern Tibetans. Proto-Tibeto-Burmans also spread over the Hengduan Mountains where the Yi have lived for hundreds of generations [28]. Taking the optimal living condition and the easiest migration route into account, we favor the single-route hypothesis; it is more likely that their migration into the Tibetan Plateau through the Hengduan Mountain valleys occurred after Tibetan ancestors separated from the other Proto-Tibeto-Burman groups and diverged to form the modern Tibetan population.

Tibetans possess biological characteristics or phenotypes unique to people who live at high altitudes. These characteristics include adaptation to hypoxia, the absence of CMS, and high offspring survival rate. Adaptation to hypoxia is mediated by the hypoxia inducible factor (HIF) complexes which consist of α (HIF-1α, HIF-2α)and β subunits (HIF-1β) [29]. *EPAS1* gene (encode HIF-2α) had undergone positive selection in Tibetans, but not *HIF-1α* despite its involvement with most of hypoxia-inducible genes [29]. *HIF-1α* is highly conserved [30] and serves as a ‘master regulator’ of cellular and systemic oxygen homeostasis [10]. Unlike *HIF-1α,* which is universally expressed [31], *EPAS1* is unique to vertebrates, neofunctionalized [32], and predominantly expressed in highly vascularized tissues such as the lung and placenta [33]. HIF-2α can escape degradation at near-normoxic conditions but HIF-1α cannot. Furthermore, unlike *HIF-1α,* which responds to acute hypoxia, *EPAS1* plays an important role in prolonged hypoxia [34], a condition with exactly the same symptoms as high-altitude hypoxia.

Another candidate gene under positive selection was *EGLN1*/*PHD2*, which is a member of the 2-oxoglutarate-dependent dioxygenase superfamily and a sensor for low oxygen levels [35]. Under normal oxygen levels, HIFα proteins are modified by prolyl hydroxylases (PHDs), resulting in the subsequent proteasomal degradation of HIF [36]. Interestingly, although HIFα stability is regulated by PHDs, PHD2 is subject to feedback up-regulation in a *HIF1α*-dependent, but *HIF2α*-independent, manner [37]. In the process of Tibetans' adaptation to high-altitude hypoxia, both *HIF-2α* and its degradation regulator *EGLN1* had undergone positive selection. However, *HIF-1α,* as the up-regulator of PHD2, had not. How exactly PHD2 reciprocally regulates HIFα requires more in-depth research.

Our gene ontology analysis showed that positively selected genes were enriched in categories related to the response to hypoxia and the development of blood vessels, embryos, and female gonads. Genes like *EPAS1* and *ELGN1*, which are involved in the response to hypoxia, may have protected Tibetans from hypoxic damage and CMS. Genes involved in blood vessel development also played important roles in high-altitude adaptation. Well-developed blood vessels can increase the efficiency of oxygen utilization. High blood flow and high infant survival rates have been two vital phenotypes that have allowed Tibetans to adapt to high altitudes [8], [38], [39]. At high altitudes, high infant survival rates are tightly correlated with heavy birth-weights [38], and are determined by placental and embryonic development. Relating to placental development, three genes, *VEGFA*, *ANGPT1*, and *ANG2*, sequentially regulate the placental vascular network from generation to maturation [40]. Our results show that *ANGPT1* was under positive selection, while *VEGFA* and *ANG2* were not. Interestingly, expression of *VEGFA* and *ANG2* can be up-regulated by hypoxia, whereas no evidence has been found that *ANGPT1* can be as well [40], [41]. In terms of embryonic development, many genes (such as *ECE1*, *TGFBR3*, *CELSR1*, and *ACVR2A*) have shown significant signs of positive selection in Tibetans. In addition, four genes (*ANGPT1*, *FSHR*, *LEPR*, and *PGR*) that are involved in female gonad development were also candidates for positive selection in Tibetans. Therefore, we can assume that positive selection acting on genes involved in the development of blood vessels, the placenta, embryos, and female gonads may have all contributed to the Tibetans' high infant survival rate.

The quality of population-based genetic studies depends strongly on unbiased sampling. This is especially important in our case, since Tibetans from different geographic regions have displayed strong heterogeneity in their genetic background (as recorded in mitochondrial genomes) [3]. Our sample individuals were from the pasture areas of Lhasa and exhibit a similar frequency distribution pattern of mitochondrial haplogroups to that of Tibetan populations from Shigatse and Nakchu in Tibet (Figure S5). Therefore, our sample collection should properly represent Tibetans living in high altitude, and yield meaningful results [11], [12].

Our data have demonstrated that the Proto-Tibeto-Burman people form a major clade within East Asian populations, and that the major migration route into the Tibetan Plateau was via the Hengduan Mountain valleys. We also found that the Yi population, and not the Han populations used in previous studies, was a more appropriate reference for exploring the adaptation of Tibetans to high-altitudes [11], [12], [13]. Moreover, in addition to previously identified *EGLN1* and *EPAS1*, we also found other potentially selected genes, including *ANGPT1*, *ECE1*, and *LEPR*, in high-altitude adaptation. These genes associate with various biological functions, such as the development of blood vessels, the placenta, embryos, and female gonads. The altered functions may certainly affect infant survival and result in adaptive phenotypes of Tibetans. More in-depth sampling and functional analyses of these genes in the future may reveal more molecular details concerning the adaptation of the Tibetan population to high-altitudes.

## Materials and Methods

### Ethics Statement

All donors signed the informed consent for cell line establishment and subsequent biological investigations. This project was reviewed and approved by the Ethics Committee at the Beijing Institute of Genomics, Chinese Academy of Sciences.

### Samples and genotyping

Thirty unrelated Tibetans (17 males and 13 females) were collected. Sample cell lines were derived from immortalization of peripheral lymphocytes by the Epstein Barr virus.. The derived cell lines were deposited at Immortalized Cell Bank of Beijing Institute of Genomics, Chinese Academy of Sciences supported by the Knowledge Innovation Program of the Chinese Academy of Sciences. All DNA samples were obtained with DNA-extraction kits (Tiangen Biotech, Beijing, China) and genotyped on the Human 1M-Duo v3 chip (Illumina, San Diego, CA, USA) according to the manufacturer's specifications. Genotyping module of Genomestudio (Illumina, San Diego, CA, USA) was employed to call the raw data. The clustering position of genotypes was determined by standard cluster file provided by Illumina. All individuals were successfully genotyped at call rate >98.1% with genotype call threshold of 0.15. We removed all CNV markers and SNPs (6,777) that cannot be accurately clustered, leaving 1,157,616 SNPs, of which 41,873 are on the sex chromosomes and 138 on mitochondrial DNA. Only autosomal SNPs were used in the study.

### Hardy-Weinberg Equilibrium (HWE)

HWE (χ^2^ test) of autosomal SNPs was tested in our Tibetan samples. Due to multiple testing, we set the threshold of the HWE test at *P* = 0.001. Of 1,115,605 autosomal SNPs, 4,066 (0.36%) failed the test and were excluded from our analyses.

### Data from public database

The Human-1M chips data of HapMap samples was downloaded from Illumina (ftp.illumina.com). Except for haplotype phasing, our HapMap-related analyses included 45 CHB, 45 JPT, 59 CEU, and 60 YRI. The HGDP data was downloaded from Laboratory of Neurogenetics (http://neurogenetics.nia.nih.gov/paperdata/public/) with 258 individuals from 14 populations: Mozabite, Palestinian, Bedouin, Druze, Balochi, Kalash, Burusho, Uygur, Yakut, Cambodian, Lahu, Yi, Daur, and Mongolian.

### Data Combination

The HapMap dataset used here was generated by Human-1M chips of Illumina and hence readily comparable to our Tibetan dataset. However, the HGDP dataset was generated by Human-550 chips of Illumina and required further data processing before use. In particular, to avoid the allele swapping problem (the complementary allele of a SNP is itself, like A/T and C/G SNPs; this will introduce an allele frequency error when combining two data sets), we removed all transversion SNPs and aligned the genotype of transition SNPs among the three datasets. In total, 509,491 overlapping SNPs between data from Human-1M chips and Human-550 chips were used for the HGDP-related analyses.

### Ancestral allele determination

The ancestral allele of a SNP was obtained from NCBI (submitted by Jim Mullikin and based on the comparison between human and chimpanzee sequences [42]). For a SNP without available information on ancestral state, the major allele of the SNP in YRI was assumed as ancestral.

### Phasing

Phasing was performed by the fastPHASE software [43] with K (number of haplotype clusters) tested from 10 to 24. For the optimal result, we used K = 14, 20, and 20 for Asian (CHB, JPT, Tibetan), CEU, and YRI, respectively. The children genotype information of CEU and YRI was included to further the phasing accuracy.

### Ancestry analysis

We inferred individual ancestry proportions with the *frappe* program [44] for 30 individuals of our Tibetan population, 497 individuals of 19 world-wide populations, and 167 individuals of 8 EA populations. To improve the computing efficiency, we removed SNPs in strong LD by the Plink software with a sliding-window approach. In a window of 50 SNPs with 5 SNPs as a step, one of paired SNPs with r^2^>0.5 was removed. Of 509,491 SNPs shared between the Tibetan/HapMap and HGDP datasets, 165,073 SNPs were retained for ancestry sharing analyses. To determine the convergence of each EM (expectation-maximization) run, either 10,000 iterations or a likelihood increase between consecutive iterations of less than 0.0001 was used for a pre-specified ancestry population number (*K*). We run at least three different seeds for each *K* from 2 to 6. Fine structures of EA populations were constructed using 8 populations. We removed two outlier populations of EA, Uygur and Yakut, for Uygur is a mixture population between EA and South/Central Asian and Yakut is a mixture population between EA and European populations [14].

### Principal component analyses

Principle component analyses were performed with the smartPCA software [45]. To observe the fine structure of EA populations, we removed the outlier of local geographic region (i.e., Yakut and Uygur was removed when analyzing EA populations).

### Phylogenic tree construction

We constructed a phylogenic tree using the *Phylip* software [46] with genotype data from 165,073 SNPs of each individual. An unrooted neighbor-joining tree was constructed with the F84 distance matrix.

### Selection tests

#### *F*_ST_ analyses

We estimated site-independent pairwise *F*_ST_ [47] between Tibetan and EA populations from HGDP and HapMap by the Genepop software [48]. With consideration of the number of SNPs per calculation and the length of extended haplotype in a selective sweep [15], the genome was divided into non-overlapping windows with 200-kb width (about 65 SNPs per window). To a window, the test statistics was calculated as the average *F*_ST_ from SNPs with *F*_ST_ value larger than 0.2 (cutoff value.) We scored zero for the test statistics of a window, if the window has no more than 3 SNPs with pairwise *F*_ST_ larger than 0.2. The empirical *P*-value of a window was obtained as the percentage of statistics greater than its window average. The window regions with *P*<0.05 were considered as candidate under positive selection. Other cutoff values from 0.2 to 0.9 had also been tested with little variation at the significant level in the windows with extremely high *F*_ST_. Larger cutoff values tend to remove the background noise of the test statistics by dropping more loci from a window. We therefore gradually raised the cutoff values to obtain the most significant (*P*<0.001) windows with extremely high *F*_ST_ in each population pair, and then assigned the empirical *P*-value to these windows. The overall windows with *P*<0.05 at the *F*_ST_ cutoff value of 0.2 for each Tibetan pair are showed in Table S2.

#### Haplotype-based selection tests (iHS and XP-EHH)

The HapMap and Tibetan datasets were used for haplotype-based selection tests. The test scripts of iHS and XP-EHH used here were provided by [15]; and normalization was performed according to [23] and [22]. As described in [15], the window size for the iHS and XP-EHH tests was set at 200 kb. In each window, we treat the fraction of SNPs with |iHS| >2 and the maximum XP-EHH as the test statistic, and converted this test statistic to an empirical *P*-value by calculating the percentage of statistics larger than that of each window. For the XP-EHH test, we used regional populations (CHB and JPT) as references for Tibetans. The overall windows with *P*<0.05 of iHS and XP-EHH for Tibetans are showed in Table S3.

### Functional annotation and clustering of GO biological process by DAVID

We generated a dataset with ten selection tests (one iHS, two XP-EHH, and seven *F*_ST_) on Tibetans. We selected the window regions significant (*P*<0.05) for at least 5 out of the 10 tests and extracted the genes from the regions as a gene list for pathway enrichment analysis. Genes with no functional annotations provided by RefSeq were removed. The final gene list (147 genes; Table S4) was analyzed by DAVID v6.7. In the functional annotation analysis, modified Fisher's exact test was used to determine the significance of gene-term enrichment with a cutoff value at *P* = 0.05. In the clustering of functional annotations, the Enrichment Score (ES) was used to rank the overall enrichment of the annotation groups. The value is defined as minus log transformation on the average *P*-values of each annotation term and was set at 1.3 (non-log scale 0.05) for significance. Additionally, a classification stringency parameter was used in the functional annotation clustering to control the fuzzy clustering of DAVID, and we used the high stringency for tight, clean and smaller numbers of clusters.

## Supporting Information

Figure S1 **Ancestry sharing proportion of samples inferred with** ***frappe*** **at** ***K*** **= 2 through to 6, using 165,073 loosely linked autosomal SNPs.** (A) for 497 individuals from 19 world-wide populations and (B) for 167 individuals from 8 EA populations.
(EPS)

Figure S2 **Principal component analyses of population structure on the 8 populations from East Asia, using 509491 autosomal SNPs.** (A) Eigenvector 2 vs. eigenvector 3. (B) Eigenvector 2 vs. eigenvector 4.
(EPS)

Figure S3 **The regions with extreme test statistics (calculated from** ***F*** **_ST_) indentified from HapMap + Tibetans sample pairs and (B) HGDP (EA population) + Tibetans sample pairs.** Each row is a 200-kb genomic window; each column is a population pair. The figure shows windows with extreme test statistics (*P*<0.001) from each population pair. The physical position of each window on the human genome was shown on the left of the figure. Gene within or near each window is showed on the right. Genes without functional summary provided by RefSeq were removed. The windows with no functional gene are not shown. Genes colored in red are well-characterized genes under positive selection in different populations.
(EPS)

Figure S4 **The regions with extreme test statistics (calculated from** ***F*** **_ST_) indentified from HGDP (EA population) + Tibetans sample pairs.** Each row is a 200-kb genomic window; each column is a population pair. The figure shows windows with extreme test statistics (*P*<0.001) from each population pair. The physical position of each window on the human genome was shown on the left of the figure. Gene within or near each window is showed on the right. Genes without functional summary provided by RefSeq were removed. The windows with no functional gene are not shown.
(EPS)

Figure S5 **The Haplogroup distribution of Tibetans with different geographic locations.** We used our genotype information from 138 mitochondrial SNP markers (included in the Human-1M chip) for haplogroup analysis, and compared our findings to that of Tibetan samples from Zhao et al (Figure R3) [3]. In short, our haplogroup's frequency distribution is similar to that of two Tibetan samples (Shigatse and Nakchu) located in the Tibetan Plateau, but is different from that of Tibetan samples outside Tibet. In particular, the frequency distributions of major haplogroups vary greatly among Tibetan populations residing outside Tibet, such as M9, F, and B haplogroups in Qinghai and most haplogroups in Yunnan. M8, M9, M10, M13, D, G, A, B, F are names of Haplogroups.
(EPS)

Table S1 **Basic information of populations under study and the numbers of markers used in different analyses.**
(DOC)

Table S2 **200-kb genomic regions with extreme high values of pairwise** ***F*** **_ST_ identified in the top five percent of empirical distributions from all Tibetan pairs.**
(XLS)

Table S3 **200-kb genomic regions identified in the top five percent of the XP-EHH and iHS tests.**
(XLS)

Table S4 **Candidate genes used for gene ontology analysis.**
(DOC)
