# Supplementary material for: On the Origin of Tibetans and Their Genetic Basis in Adapting High-Altitude Environments
Source: PLoS One. 2011 Feb 28;6(2):e17002. doi: 10.1371/journal.pone.0017002 (PMC3046130; doi:10.1371/journal.pone.0017002)
Supplement: Table S1 — Basic information of populations under study and the numbers of markers used in different analyses. (DOC) [file pone.0017002.s006.doc]

Table S1. Basic information of populations under study and the numbers of markers used in different analyses

| Abbreviation | Population | Continent/Area | No. of individuals | Data deposition | geographic distribution in East Asia | No. of Markers used in | | | | | | |
| --- | --- | --- | --- | --- | --- | --- | --- | --- | --- | --- | --- | --- |
|  |  |  |  |  |  | *frappe* | fastPHASE | smartPCA | *Phylip* | *F*_ST_ | iHS | XP-EHH |
| YRI | Yoruban | Africa | 60 | HapMap | - | 165073 | 1111539 | 509491 | 165073 | 1111539 | 1111539 | 1111539 |
| Mozabite | Mozabite | Middle East | 30 | HGDP | - | 165073 | - | 509491 | 165073 | 509491 | - | - |
| Palestinian | Palestinian | Middle East | 26 | HGDP | - | 165073 | - | 509491 | 165073 | 509491 | - | - |
| Bedouin | Bedouin | Middle East | 47 | HGDP | - | 165073 | - | 509491 | 165073 | 509491 | - | - |
| Druze | Druze | Middle East | 43 | HGDP | - | 165073 | - | 509491 | 165073 | 509491 | - | - |
| CEU | Utah residents with Northern and Western European ancestry | European | 59 | HapMap | - | 165073 | 1111539 | 509491 | 165073 | 1111539 | 1111539 | 1111539 |
| Balochi | Balochi | South/Central Asia | 15 | HGDP | - | 165073 | - | 509491 | 165073 | 509491 | - | - |
| Kalash | Kalash | South/Central Asia | 18 | HGDP | - | 165073 | - | 509491 | 165073 | 509491 | - | - |
| Burusho | Burusho | South/Central Asia | 7 | HGDP | - | 165073 | - | 509491 | 165073 | 509491 | - | - |
| Uygur | Uygur | East Asia | 10 | HGDP | - | 165073 | - | 509491 | 165073 | 509491 | - | - |
| Yakut | Yakut | East Asia | 15 | HGDP | - | 165073 | - | 509491 | 165073 | 509491 | - | - |
| Cambodian | Cambodian | East Asia | 10 | HGDP | south | 165073 | - | 509491 | 165073 | 509491 | - | - |
| Lahu | Lahu | East Asia | 8 | HGDP | south | 165073 | - | 509491 | 165073 | 509491 | - | - |
| TIB | Tibetan | East Asia | 30 | our data | southwest | 165073 | 1111539 | 509491 | 165073 | 1111539/509491 | 1111539 | 1111539 |
| Yi | Yi | East Asia | 10 | HGDP | southwest | 165073 | - | 509491 | 165073 | 509491 | - | - |
| Daur | Daur | East Asia | 10 | HGDP | north | 165073 | - | 509491 | 165073 | 509491 | - | - |
| Mongolian | Mongolian | East Asia | 9 | HGDP | north | 165073 | - | 509491 | 165073 | 509491 | - | - |
| CHB | Han Chinese | East Asia | 45 | HapMap | north | 165073 | 1111539 | 509491 | 165073 | 1111539 | 1111539 | 1111539 |
| JPT | Japanese | East Asia | 45 | HapMap | northeast | 165073 | 1111539 | 509491 | 165073 | 1111539 | 1111539 | 1111539 |
