# Supplementary material for: On the Origin of Tibetans and Their Genetic Basis in Adapting High-Altitude Environments
Source: PLoS One. 2011 Feb 28;6(2):e17002. doi: 10.1371/journal.pone.0017002 (PMC3046130; doi:10.1371/journal.pone.0017002)
Supplement: Table S4 — Candidate genes used for gene ontology analysis. (DOC) [file pone.0017002.s009.doc]

Table S2. Candidate genes used for gene ontology analysis

| Genes analyzed by DAVID | | | | | | |
| --- | --- | --- | --- | --- | --- | --- |
| *A2M* | *CELSR1* | *EIF2A* | *HLA-DQA1* | *MAT2B* | *PDGFC* | *RYR1* |
| *ABCC4* | *CHGA* | *ELSPBP1* | *HLA-DRB1* | *ME3* | *PGR* | *SAFB* |
| *ACCN1* | *CLEC2D* | *EPAS1* | *HLA-DRB5* | *MRPL39* | *PIGF* | *SCN2A* |
| *ACTN1* | *CLECL1* | *EPB41* | *HPSE2* | *MRPS9* | *PITX1* | *SELT* |
| *ACVR2A* | *CLN5* | *ERC1* | *IL10RB* | *MYC* | *PKP2* | *SEMA6D* |
| *AGTPBP1* | *CNTN5* | *FANCL* | *IMMP2L* | *NELL2* | *PLA2G4C* | *SFRS4* |
| *AMPD2* | *CNTNAP2* | *FOXO1* | *IPO13* | *NKAIN2* | *PPFIA2* | *SGCZ* |
| *ANGPT1* | *CNTNAP5* | *FSHR* | *ITSN1* | *NPAS3* | *PREX1* | *SLC8A1* |
| *AQP7* | *CRIM1* | *GLIS3* | *KCNA4* | *NRG3* | *PRIM2* | *SLCO1B3* |
| *ARNTL* | *CYLC2* | *GNAT2* | *KCNIP4* | *NRP2* | *PRKCE* | *SLITRK6* |
| *ASB3* | *CYP7B1* | *GNPAT* | *KIRREL3* | *NRXN3* | *PRSS23* | *SMARCD3* |
| *ATP5O* | *DCC* | *GOLGA5* | *KLHL1* | *NUDT12* | *PSMD9* | *SMG6* |
| *BCL7A* | *DISC1* | *GPR61* | *KLRG1* | *OLFM4* | *PTPDC1* | *SOCS5* |
| *CABP5* | *DLG2* | *GRIA1* | *LCORL* | *OR6N1* | *PTPRD* | *SVIL* |
| *CC2D2A* | *DNAH5* | *GRID2* | *LEPR* | *OTOF* | *PTPRN2* | *TGFBR3* |
| *CD69* | *DOK6* | *GRIN2B* | *LIG1* | *PARD3* | *RAB28* | *TMPRSS2* |
| *CDH12* | *DPH1* | *GRM7* | *LPHN2* | *PCDH15* | *RGNEF* | *TSNAX* |
| *CDH13* | *DPH1-OVCA2* | *GRM8* | *LPHN3* | *PCDH20* | *RHOQ* | *WARS2* |
| *CDH18* | *ECE1* | *GSTM4* | *LRP1B* | *PCDH7* | *RPL10L* | *WWOX* |
| *CDH8* | *EDIL3* | *H2AFY* | *LRRC4C* | *PCDH9* | *RUNX1* | *XPNPEP1* |
| *CDH9* | *EGLN1* | *HAS2* | *MAP2K5* | *PDE4B* | *RUNX3* | *ZEB2* |
